# Copy of Reports and Communications on M. Ledoyen's Disinfecting Fluid

**Published:** 1847-10-01

**Authors:** 


I. Copy of Reports and Communications on M. Ledoyen's Disinfecting Fluid, with Supplement. Ordered by the House of Commons to be printed, 1st July, 1847.

II. Copy of Reports on Sir William Burnett's Disinfecting Fluid. Ordered by the House of Commons to be printed, 20th July, 1847.

In noticing these two Parliamentary papers, we must protest *in limine* against the very objectionable misapplication of the term "disinfecting," as employed in both. A vast deal of error and confusion has been introduced into medical reasoning by the vague and unsettled use of the words infect, infection, disinfecting, and so forth. Every one, we presume, will admit that they should all have a correlative signification, and that this signification should be definite and unfluctuating. In recent numbers of this Journal, we have sought to attain this important end by limiting the term "infection" to denote that power or quality of certain maladies to propagate themselves by effluvia given off from the bodies of the sick, and becoming diffused through the surrounding atmosphere. It must be quite obvious that, unless some such definitive restriction be adopted, it will be utterly impossible to argue with any degree of intelligible precision, far less to determine with scientific accuracy many of the questions connected with the important subjects of the origin and dissemination of febrile diseases, as well as of the best means of checking their growth or arresting their progress. We are well aware that many medical writers, and those too of high repute, have used the words in question in a larger and more comprehensive sense than that which we think wise. Still, without almost any exception, they have very generally been employed in reference to the production and presence of morbific effluvia. It is for this reason that we object so strongly to the employment of the term "disinfecting" in the sense of "stench-destroying," or even of "putrefaction-arresting;" and we therefore cannot but regret that Sir William Burnett should have given the sanction of his high authority to the perpetuation of such a misnomer,—calculated, as it is, to convey an erroneous impression to the public. It is not at all to be wondered at that M. Ledoyen, and the great trumpeter of his nostrum, Colonel Calvert, should have fallen into the blunder; if *blunder* it can be called in their case, seeing that they claim for their fluid the property not only of destroying all noisome smells, but even of "disinfecting patients suffering with infectious disorders." We shall presently see what evidence or proof can be adduced for this assertion. When M. Ledoyen, or rather Col. Calvert in his name, applied to the British Government for assistance in order that (to use his own modest words) "this country may, by publishing it here in Great Britain, spread the beneficent works of the Creator, who has given by his wisdom to man this important discovery to prevent disease," Lord Morpeth very wisely committed the investigation of the said "extraordinary discovery" (for so it is termed) to Dr. Southwood Smith and to Messrs. Grainger and

Toynbee. These gentlemen have drawn up an elaborate report upon the subject: we now proceed to give a summary of its chief contents.

From their examination and experiments, there can be no doubt that the Ledoyen fluid is an excellent corrector of foul and noisome stenches. So far, then, it may become a most useful application in a variety of circumstances, which need scarcely be mentioned. The trials, that were made with it for the purpose of counteracting the offensive odour of fæculent and other discharges in the wards of hospitals, &c., were altogether most satisfactory. To nightmen too, and others engaged in the offensive, and often not unperilous, task of emptying old cesspools, opening foul drains, &c., the free use of the fluid will be found of the very greatest service. So far the report is altogether favourable to the discovery. Let us now see what other properties it possesses.

Although powerfully *anti-bromic** (to use an epithet suggested by Dr. Macdonnell, who wisely discriminates between the properties of "stench-destroying" and of "disinfecting), the Ledoyen fluid does not possess, it would appear, any very notable *antiseptic* properties.

"The result of experiments," say the reporters, "induces us to doubt whether this fluid be more efficacious in checking the process of decomposition than other methods already known and in common use. As to large and solid parts, such as a whole limb, it is certain that they cannot by mere immersion in this fluid be kept in a state fit for scientific purposes. Membranous organs, as the intestine, may be somewhat better preserved by this fluid, and the same may be true of their sections of solid organs, as of the liver; but discolouration occurs in all these cases, and putrefaction is but imperfectly prevented. It is the opinion of a person thoroughly versed in these matters, and who assisted us in making these examinations, that a strong solution of nitrate of potash mixed with a very small portion of corrosive sublimate (bichloride of mercury), a preparation long and extensively used, is in all respects as effective as the fluid of Mr. Ledoyen.

"We are therefore of opinion, that whilst this fluid may be regarded as a useful addition to the preparations already known for preserving parts for dissection, it has no peculiar efficacy in the preservation of the dead body. Still, however, from the result of experiments hereafter to be stated, we have no doubt that this fluid might be usefully employed in the dissecting-room, to disinfect the atmosphere, and even for the removal of fætor from the exposed surfaces of parts under dissection." P. 7.

Having thus ascertained the qualities of the fluid as an antibromic and antiseptic, we have next to examine whether it be entitled to the appellation of a "disinfectant," in the sense of a corrigent or antidote to the operation of infectious or disease-transmitting effluvia.

Colonel Calvert, indeed, takes this point for granted; for he does not hesitate to assure Lord Morpeth that "it disinfects sailors suffering from fever on board of vessels; it will also disinfect ships at sea and under quarantine. It disinfects patients suffering with infectious disorders and wounds, also dead bodies, so that they may be kept nearly a month." The jumbling together here of conditions, which have scarcely any analogy with one another, can only be excused on the ground of the Colonel's knowing very little of what he was writing about. As to the preservative

---

* Αντι, *against*, and βρωμος, *fœtor*.

effects of the fluid, we have already seen how far the evidence of the reporters warrants his very confident statements. But what say these gentlemen on the much more important allegation as to its alleged power of counteracting the agency of infection ? At page 11 we read thus :—

"In severe and malignant fevers, especially when several cases of this character occur at the same time, and when the wards of the hospital are crowded, as always happen when fever becomes epidemic, the nurses invariably suffer, and some of them are sure to perish. In our opinion the use of this fluid will be a great protection to this class of persons under such circumstances, while, by keeping the air of the wards fresh and wholesome, it will be alike protection to the physicians and other officers, as well as to the friends of the patients who come to visit them, and at the same time it must operate favourably on the general course of the fever itself.

"There is another important application of this fluid in fever, and in all other diseases in which the excretions of the body become vitiated. It rarely happens at the fever hospitals, that a laundress who retains her situation a few months, when fever is at all prevalent, escapes an attack of fever ; in bad cases of fever the evacuations are passed in bed, involuntarily and unconsciously, and all the excretions of the body with which the bed-clothes become saturated are much more noxious than in health. The effluvia which arise from these bed-clothes, when they are washed, are sure, sooner or later, to produce fever in those who inhale them. By immersing the bed-clothes and the body-linen in this fluid, it is probable that many lives might be saved, and that the constitution in still more instances might be prevented from receiving a shock which is never recovered, even when death is not the immediate result of the attack. It is obvious that the same must be true in other diseases besides fever; in fact, wherever the secretions and excretions become vitiated." P. 11.

It will be observed that the opinion here expressed is given only as a probable conjecture, and not as the result of any satisfactory experiments. The correction of fætid effluvia is too readily assumed as a ground for believing that there is, at the same time, a necessary neutralisation or destruction of infectious miasms. But this is the very fact we want to discover. The reporters seem to have taken for granted that, if the one be the case, the other must be so likewise. At least, their language fairly bears this interpretation ; for, in the very next sentence after that just quoted, it is remarked :—

"With reference to the power of this fluid to restore *purity to contaminated air*, it is important to advert to the common practice of burning pastiles and other matters to remove offensive odours from sitting and other rooms. The effect of this practice is merely to overpower an offensive by an agreeable odour; the cause of the offensive smell still remains in full operation, only our senses are prevented from appreciating it; but when, on the contrary, the fætor of a room is removed by this disinfecting fluid, the gas upon which the fætor depends is decomposed, and therefore the air of the room is purified by the removal of the very cause that contaminated it." P. 11.

The expression which we have marked in Italics, occurring in the course of observations upon the spread of "severe and malignant fever," must naturally be regarded by most medical readers as implying an allusion not merely to a fætid or offensive, but to a positively infected or miasm-charged, condition of the atmosphere. Moreover, that Dr. Smith (for we may reasonably suppose that he was the writer of this part of the Report) is constantly associating in his mind the co-existence of foul smells with the generation and diffusion of infectious fevers is pretty obvious, from the circumstance of his making the remark, immediately after alluding to the promptly-fatal effects of Sulphuretted Hydrogen in a concentrated state upon human beings, that " we trace its remoter consequences in the fevers and choleras that follow." Yet, as in very contradiction of this mutual association, we find that Dr. Leeson,—to whom the Reporters had applied for his opinion as to the virtues of M. Ledoyen's fluid—in his tabular arrangement of putrescent animal and vegetable effluvia, has classified " typhoid miasmata" among those which are *dangerous but inodorous*; and he (Dr. L.) adds, in reference to the fluid :—

" In regard to its efficacy as a *disinfecting agent,* the general result of these experiments establish the conclusion that the efficacy of this process is confined to the removal of the unpleasant odours due to sulphuretted hydrogen and hydro-sulphate of ammonia. As the sulphuretted hydrogen is the most abundant and most offensive of the various products of animal and vegetable decomposition, it is evident that, although this process cannot remove the whole of the offensive odours, it is still well suited to effect a very important and extensive amelioration of the nuisance arising therefrom." P. 13.

We may here state that the fluid in question is a solution of the Nitrate of Lead. Besides the advantage of being inodorous itself, it possesses this not inconsiderable one, viz., that the nitric acid combines with the ammonia of the putrescent matters, thus preventing its dissipation, and forming at the same time a salt which is known to be highly serviceable for agricultural purposes. Whether the metallic sulphuret, that is formed at the same time, may prove at all hurtful when the soil is dressed with manure that has been previously treated with the fluid, remains to be found out. The antibromic properties of the preparation are entirely owing to its power of fixing and neutralising sulphuretted hydrogen gas and hydro-sulphuret of ammonia. That it has any power in destroying other sorts of stenches appears to be doubtful. It is to be regretted that the reporters did not think of comparing it with other preparations which have been used for the same purpose, before going so far as to recommend to the Government, "that, as it renders the removal of night-soil practicable without creating a nuisance, it ought, in our opinion, to be made a matter of police regulation that no privy or cess-pool should be emptied without the previous use of a sufficient quantity of *it* to destroy all offensive smell." After the word " it," would it not have been only fair to insert, " or of any other equally effectual counteragent?" In making this remark, we allude more particularly to Sir William Burnett's (improperly designated) " disinfecting fluid," which has been much longer before the public than that which Colonel Calvert attempts to thrust upon us with such intemperate zeal. As strong and very conclusive evidences of the exceedingly useful properties of the Burnett fluid, we shall select two or three of the certificates which are adduced in its favour, in the Parliamentary Report just published, so that the reader will be able to form a tolerable fair estimate of the comparative advantages of the two preparations:

" Royal Naval Hospital, at Haslar,

12 July 1847.

" Sir,

" In compliance with your directions to us to report on the use of Sir William Burnett's fluid *as a disinfectant, or as to the removal of noxious smells,* we have to inform you that it has been used in this hospital in the close-stools of patients affected with dysentery, in the water-closets and cesspools, and also in the wards, when the air was tainted by purulent expectoration or discharge from sores, with the effect of immediately removing the disagreeable odours. It has also been used in the surgery with good effect, in removing the smell of putrefying animal substances, the odours of dead bodies under inspection, and when employed as a dressing to ulcers, it removes the disagreeable smell of purulent matter, and in the proportion of one part of the clear solution to eighteen of water, it preserves subjects of natural history from putrefaction, and in a fit state for anatomical inspection, after more than a year has elapsed, or as long as our trials of it have lasted. We have had no contagious or epidemic diseases in the hospital, by which its powers of arresting infection might be tested; but it has been used, much diluted, for sponging the skin of patients affected by fever, with evident benefit, and the immediate removal of the odour of perspiration, and as *it is itself inodorous*, it is in no way offensive to the patients.

We have, &c.

(signed) *John Richardson*, Medical Inspector.

(signed) *J. Anderson*, Medical Inspector.
*James Allan*, Deputy Inspector.
*Alexander M'Kechnie*, M.D. Surgeon.
*Alexander Stuart*, Assisting Surgeon."

Captain Superintendent

Sir W. E. Parry.

The fluid has been most extensively used for the correction of the noxious effects of foul bilge-water, and with the most satisfactory results, as evidenced by the reports of various naval surgeons, captains, and ship-builders. One will suffice. Mr. Chapman, the surgeon of H. M. S. "Porcupine," says:

"I have to inform you that your solution has been applied to the holds of the 'Porcupine,' (in which vessel I have the honour to serve), and although a very short time has elapsed since its application, the disgusting effluvium which previously existed is now entirely removed; indeed, its effects appear to be instantaneous, for, on the morning after applying the solution, not the slightest fætor existed; and, as a further proof of its perfect success, plate, gold lace, and other metallic substances, now retain their colour and brilliancy, which before could never be kept from tarnishing—evidently showing the corrosive principles are also removed; but, above all, the health of the ship's company has already improved, and their comfort has been much enhanced by the removal of the deleterious and highly offensive effluvia which were emitted previous to the application." P. 9.

Mr. Bowman testifies to "its value as a preservative of animal structures prepared by the anatomists. When used in a proper degree of dilution (about one part to fifty of water), its success is complete, and it appears to me to preserve the colour and texture of the parts very admirably. It has the further very important advantage of not acting on the steel instruments, being, in this respect, equal to alcohol." Equally conclusive is the evidence of Dr. Sharpey, and other gentlemen.

Professor Quain has used the fluid as an application to sloughy tumours, &c., and he is of opinion that "it will supplant the Chloride of Lime and Soda altogether in the removal of fætid odour." The only evidence, by the bye, adduced in favour of the Ledoyen fluid for this purpose, is a somewhat unsatisfactory communication from Mr. Travers, junior, relative to one case of "fætid and ill-conditioned sores" on the leg, in which it was applied at St. Thomas's hospital.

It would seem, therefore, that Sir W. Burnett's preparation is vastly superior to M. Ledoyen's as an *antiseptic*; while, at the same time, it is equally potent as an *antibromic*. Let us now see whether there is any evidence of its (the former) being a disinfectant,—in other words, possessed of any power to arrest or modify the spread of infectious diseases. What thinks the reader of the following communication from Mr. Varling, the surgeon of H.M.S. "Vengeance," to Sir. W. Burnett?

"Having used the chloride of zinc rather extensively on board Her Majesty's Ship 'Vengeance,' whilst employed in the conveyance of troops, I think proper to report to you the result thereof. We carried the 1st battalion of the 42nd regiment, consisting of about 700 men, women, and children, from Malta to Bermuda. Measles had prevailed epidemically in the regiment previously to their embarkation, but we received none on board labouring under the disease; yet, after being ten days at sea, several cases occurred simultaneously among the soldiers, and, on the 1st of April, having been then a month at sea, the disease appeared among our own people, ten cases occurring on that day, and from that day to the 15th of the month, when we arrived at Bermuda, fresh cases were almost of daily occurrence, either among our own people or the troops. On getting rid of the troops, which we did at Bermuda, my attention was of course specially directed to every means whereby the contagion could be destroyed. Cleanliness and ventilation were duly attended to, and every part of the ship where the sick had been, after being cleaned and aired, was sponged well over with the solution of chloride of zinc several times. Than the result, nothing could be better; the disease totally ceased, no fresh case occurring after. On our passage from Halifax, with the 60th regiment on board, the weather was so bad, and the ship working so much, that it was quite impossible to open any of the lower-deck ports, on which deck the whole of the people lived, troops as well as our own people, for eight days; the air throughout the deck was exceedingly vitiated with every mixture of noxious smell, but the free use of the chloride of zinc tended, in a most surprising manner, to do away with the bad smell; so much so, that the surgeon of the regiment came to me to get some to use in the part of the ship where the ladies of the officers were. The effect of the chloride of zinc is most obvious in correcting all bad and offensive effluvia; and from the sudden and surprising manner in which the measles disappeared after its use, it is not, I think, too much to say, that it must have been very instrumental in decomposing the miasm, or state of atmosphere in the ship, which tended to the generation of the disesse." P. 12.

Does the evidence warrant Mr. Varling's conclusion? We think not. Perhaps the following statement from Dr. Cronin, as to the effects of the solution when freely used in the fever hospitals at Cork, may be deemed a little, but only a little, more satisfactory:—

"I commenced," says Dr. C., "its general and exclusive use on Sunday the 20th June 1847, chloride of lime having been previously in use, and have no hesitation in distinctly stating, that I found it a powerful agent in speedily removing all noxious, unwholesome, and offensive smells, arising both from the external and internal excretions of a number of persons occupying the same room, and which will arise, no matter how well soever ventilated such apartments may be.

"This effect of the use of chloride of zinc was not only observable in all the wards of both hospitals, but in the yards, necessaries and cesspools.

"That the purification of the air should have much influence in modifying the character, and in mitigating the severity of a disease (by many, very many, supposed to be contagious), is an axiom very extensively, if not generally, admitted.

"How far such influence has been exercised by the use of the chloride of zinc on the cases of disease under treatment, during the time before specified, I leave to you and others to draw your and their own conclusions from the facts which I am now about to state.

"In the month ending June 15th, 83 persons of both sexes were treated in hospital; of those, 10 died. Whereas, from June 15th to this day, July 15th, there were admitted 162, of those four died (three males and one female), 94 were discharged cured, and 64 remain under treatment.

"Of those who died in this latter month, one was a very old man, a long confirmed drunkard; one a boy of cachectic constitution from infancy, and one a woman with pneumonic complication.

"You have seen how many (almost all) of those cases were of a severe typhoid character." P. 12.

Dr. Lindsay, Deputy-Inspector of Hospitals, in transmitting Dr. Cronin's letter, remarks:

"The patients admitted into the Cove Hospital are mostly all labouring under fever of a typhoid form, many of them with the characteristic eruption at the beginning, and petechiæ appearing during the progress of the disease, accompanied by great debility, feeble pulse, and all the other symptoms of bad typhus.

"The rate of mortality, however, has been exceedingly low since the use of the chloride of zinc was commenced, and if any conclusion can be drawn from a three weeks' trial of it as a disinfecting agent, it must be highly in its favour.

"I herewith enclose a report from Dr. John J. Cronin, the present attendant physician of the hospital, and as I have myself been in almost daily observation of its use amongst his patients, I can add my testimony to his." P. 13.

We have already seen what the reporters on M. Ledoyen's fluid have stated on this point. Their report, it deserves to be noticed, is dated 29th March of the present year. On the 27th of April, they addressed the following letter to Col. Calvert.

"There is nothing in Mr. Ledoyen's disinfecting fluid that can arrest the progress of fever, or influence the connexion which the history of the human family shows is universal and indissoluble between pestilence and famine. But though this preparation can produce no effect on the primary, it may have some influence on the secondary causes of fever, that is on those causes which increase its intensity, and favour its spread when once generated.

"Among the most powerful of the secondary causes are the excretions of the patients, whether those from the bowels, the lungs, or the skin. The disinfecting fluid will instantly remove all putrescent smell from uterine (alvine?) discharges, leaving only the odour of recent fæces, and that in a less offensive degree, and at the same time it will render those discharges very much less capable of giving off their volatile and diffusible exhalations. It is also probable that it may be so used as to keep the air of the hospital ward and of the private sick chamber in a state of purity, by destroying the offensive odours arising from the exhalations of the breath and skin.

"It is therefore our opinion, that it would be useful to make this preparation known to the physicians and surgeons of Ireland, and also to private families; for if it be properly and assiduously used, it may have a beneficial effect upon the sick themselves, by keeping the air around them pure, and thereby materially facilitating their recovery, and it will unquestionably be a great protection to the attendants on the sick. But we must beg leave to give a distinct caution against this preparation being spoken of as a remedy in fever, for if it be so considered, it must lead to disappointment, and it will probably bring doubt on the properties which it really possesses, and which are capable of being applied in various ways to the public advantage." P. 35.

Dr. Leeson also, at the same time, having been requested by Col. Calvert to give his opinion as to what might be expected from the use of the fluid "in arresting the progress of infection and fever," expresses himself to the following effect:

"Until much more is known with regard to the true exciting cause of fever, or, rather, the immediate vapour exhalation or gas by which such infection is conveyed, it would be presumptuous in any one to say whether such cause would or would not be affected by your liquid.

"This, then, is an important object of experiment.

"It is well known, that wherever a fever-exciting atmosphere does exist, there also abound the very gases which your liquid is capable of destroying; but whether such gases are merely concomitant, or whether they are themselves wholly or partly the exciting cause, is at present a mystery: this, at least, is evident, that such concomitants are not harmless, although (as shown in the paper already referred to) the general opinion seems to incline to the belief that there are other exhalations which are the true miasmata on which fever is dependent." P. 36.

On the 26th of May, we find Dr. Smith writing to Col. Calvert respecting the use of the fluid in the Fever Hospital here, in these terms:—"It is nearly full, and we find the use of the fluid in the present crowded state of the wards extremely beneficial." He had previously remarked in the same letter;—"that there should be no death in a ward in which six deaths occurred daily before the disinfecting fluid was used, is an amount of success, if it should continue, which was not to be expected, and the continuance of which is not to be looked for." This certainly leaves a very favourable impression as to the "disinfecting" properties of the fluid on the mind of an unprofessional reader at least; we cannot, therefore, be surprised that Colonel Calvert pounces upon the Doctor's letter with peculiar avidity:—"it must excite," says he, with his usual *fanfaronade*, "every feeling of humanity to get this fluid into immediate general use, *as it will save the lives of thousands who are at this time afflicted with fever and dysentery*."

A month subsequently to the date of this propitious letter, viz. on the 28th of June, appeared another letter from Dr. Smith, which, not only as it contains an account of his further experience, but also as it enunciates opinions of very questionable accuracy, deserves particular notice: It is addressed to Lord Morpeth.

"My Lord, 28th June, 1847.

One of the constant and distinguishing characters of a severe epidemic is that it attacks the attendants on the sick. The fever which is at present prevailing to such a deplorable extent in almost every part of the United Kingdom exhibits this character in an unusual degree. From the accounts daily received from the larger towns in England, but particularly from those of Ireland and Scotland, it is certain that in great numbers of instances fever is communicated not only to clergymen and relieving officers who visit the sick in their own wretched homes and poisonous localities, but also to the nurses and medical men in attendance even on private families; while it is far more prevalent and mortal among nurses, medical students, and the surgeons and physicians of hospitals and unions. Now this part of the calamity at least might be spared. Whatever difficulties your Lordship may have encountered in obtaining the necessary powers to make even any commencement of a system of prevention, by the removal of the causes of fever, you have in your own hands, and have had for some months past, the sure and certain means of preventing the extension of fever to the immediate attendants on the sick. An agent has been discovered (M. Ledoyen's Disinfecting Fluid) capable of entirely destroying the noxious gases arising from decomposing animal and vegetable substances. The properties and powers of this fluid, after having been examined by a series of careful and exact experiments, performed partly under your Lordship's own observation, have been further tested in the crowded and poisonous fever-wards of the hospitals and unions of Manchester, Liverpool, and Dublin. All classes of witnesses, from the nurses and wardsmen to the highest medical authorities, without a single exception, have corroborated (from what they have themselves seen) the correctness of the conclusions deduced from the original experiments, and given in detail in a report presented to your Lordship on the 29th of March, 1847.

"When used in a sick chamber, or in hospital and union wards, this disinfecting agent decomposes and destroys the poisonous matters given off from the breath and skin, and from all the discharges of the body, and thus maintains the air surrounding the patients in a state of perpetual purity. It therefore effects more than ventilation; for while ventilation merely dilutes the poisonous matters diffused in the air, by the introduction of fresh currents of pure air, this agent destroys the very *sources* of impurity.

"No instructed person will suppose that this fluid can exercise, as a remedial agent, any influence on the state of fever itself, or on the diseased processes so often set up in it; yet the effect produced indirectly by it (merely by maintaining the purity of the surrounding air), in improving the condition of the patient, is sometimes most striking and permanent. It is a further property and advantage of this fluid, that it creates no disagreeable odour of its own (as is the case with other disinfecting agents), but, on the contrary, produces a peculiar sensation of freshness.

"I have been unable to afford my patients in the Fever Hospital the full benefit of this important discovery, on account of my inability to procure the fluid in sufficient quantities for daily and regular use. I have regretted this the more, because a bad form of erysipelas, proving fatal in several instances, has spread extensively through the wards, and I am satisfied that this might have been checked by the free use of this fluid.

"I have also been anxious to procure enough of the fluid to immerse in it the body-linen and the bed-clothes of the patients; for we have scarcely ever had in the Fever Hospital a laundress who has not sooner or later been attacked by fever; but, from what has been stated, it is obvious that all these classes of persons, nurses, laundresses and medical men, who are always in imminent danger, and who so often suffer, might perform their arduous duties with perfect security. I therefore respectfully but earnestly beg of your Lordship no longer to withhold from the public, more especially in the present condition of the country, the knowledge of a preventive and remedial agent, the general employment of which (irrespective of other uses to which it is applicable) will undoubtedly contribute towards saving the lives of many valuable persons." P. 24.

That any instructed medical man should entertain so high an opinion of this, or of any other, alleged disinfecting agent as unwaveringly to assert that "it decomposes and destroys the poisonous matters given off from the breath and skin," and also that he has in his own hands *the sure and certain means of preventing the extension of fever to the immediate attendants on the sick*, does certainly surprise us not a little; and we must withhold our belief in the accuracy of such assertions, until much more satisfactory evidence be adduced than what has yet been brought before the public. Would it not have been well to have given the *ipsissima verba* of the medical officers of the fever hospitals alluded to, touching the very important point in question? And is it not rather surprising that Dr. Smith, on the very day that he addressed Lord Morpeth in the terms we have just read, wrote to Colonel Calvert, respecting his own personal experience of the fluid, in language that is much less energetic and unqualified?

"By the free use," says he, "of the disinfecting fluid in the bed-pans; by the suspension of cloths saturated with it round the beds of the patients, and by the abundant diffusion of its vapour in the atmosphere of the wards, I am satisfied that the poisonous exhalations constantly emanating from the bodies of the patients *would have been* decomposed and destroyed much more rapidly and completely than could have been effected by any amount of ventilation alone." P. 26.

And straightway he adds this not unimportant statement respecting the very salutary effects of simple Ventilation on fever patients:—

"By the construction and arrangement of the windows we have it in our power to introduce into the wards of the fever hospital any quantity of fresh air we desire; and the soothing and healing influence of this air on the patients is most striking; for when brought into the hospital in a state of violent delirium, with a parched and black tongue, they often become perfectly calm, and the tongue gets moist and begins to clean at the edges in a few hours after they have breathed the comparatively cool and pure air of the spacious and well-ventilated fever ward; but surely we may hope to effect still more, when, in addition to the inestimable advantage of ventilation, we obtain the means of destroying at their very sources the impurities which it is the object of ventilation to remove." P. 26.

The rest of the letter about the doctor and his family eating the peas and potatoes, which had been raised in his garden that was well manured with *Ledoyenised* night-soil, without suffering from any symptoms indicative of lead or of any other poison, need not detain us.

In place of the frivolous communications from the resident medical officer of the Fever Hospital at Battle Bridge, how comes it that Dr. South's colleague has not been applied to, to give his opinion of the virtues of the marvellous fluid? A few lines from Dr. Tweedie would surely have had greater effect, with the profession at least, than the correspondence between Mr. Sankey and Colonel Calvert. By-the-bye, we accidentally learn from Mr. Sankey a strange and certainly very reprehensible state of things that occasionally exists at the fever hospital, viz. that dead bodies have sometimes to be kept eight or ten days in the dead-house, "owing to the clumsy working of the registry of deaths."

The evidence of the resident medical officers of the Liverpool Fever Hospital merely goes to prove the stench-destroying properties of the fluid; but these gentlemen say not a word as to its efficacy as a "disinfectant." The same thing may be said of the testimony of the medical officers of the Infirmary and other hospitals at Liverpool. We now pass over to Dublin, where the Colonel seems, according to his own report, to have achieved his greatest triumphs. His modest statement is as follows:—

"On our arrival (in Dublin), I had the honour to present Mr. Labouchere's letters to Mr. Redington and Sir P. Crampton, one from Sir W. Somerville to Mr. Carmichael, and your Lordship's kind letters to Mr. Macdonnell and Mr. Roe: I was most kindly received, and immediately assisted by all to put to the test of the fluid in the fever and dysentery wards of different hospitals, and a most dreadful dysentery ward was selected by us to show what could be effected: it was, in fact, the condemned ward of the North Union Workhouse, Dublin, where poor wretches were sent to die, and they usually had five or six deaths a week: effluvia and the stench was dreadful; the men in a wretched and helpless state: the *doctors refused going into it:* in the presence of at least a dozen physicians and surgeons, we entered; we undertook the care of the ward and patients; we purified it in a very short time; we recovered the sick, and we did not lose a single patient during three weeks: this will be certified to your Lordship by certificates from the physicians, &c. of the house, and others who witnessed, to their astonishment, this severe test: we have had other severe tests, for all of which I hold certificates from the physicians, &c."* P. 36.

We beg, before proceeding, to say that we do not believe the assertion contained in the words we have italicised. And now, how is the above statement born out by the medical certificates? Sir P. Crampton testifies to the *antibromic* properties of the fluid, and to them alone. Sir H. Marsh and Dr. Cusack certify to its effects "in quickly neutralising and finally destroying the noxious effluvia arising from urinary and fæcal deposits." Dr. Carmichael, while recognising its value in destroying fætid odours, says:—"As to its power of disinfecting contaminated places, I could not positively vouch, without an extended system of experiments fairly conducted; but I think there is a strong presumption that it does possess this power, from the fact that, while it removes the most offensive odours, it leaves none in their place."

Dr. Kirkpatrick, also, physician to the Workhouse where the wonderful effects were produced, after mentioning certain facts shewing the efficacy of the fluid in "decomposing, or in some unknown manner of removing, noisome odours, and without producing any peculiar smell of its own," observes:—"The patients experienced great relief from the improvement in the air; and it is strange, although several deaths had occurred in this ward during the preceding week, yet, during the time of his visit here, no death happened amongst the patients first seen by Colonel Calvert," and on the very following day, when writing to the Colonel, Dr. K. adds:—"The extraordinary effect it possesses in the decomposition of foul odours has been proved beyond dispute, and that to it may also belong the greater value of destroying that pernicious condition of the atmosphere upon which disease depends is neither impossible nor improbable."

There is another communication from Dr. Kirkpatrick, which for his sake, we trust, was never intended for publication; as it is anything but creditable either to his judgment or good taste. It contains, we may observe, nothing that is worthy of notice. Not so the letter of Drs. Carmichael and Macdonnel; who, in alluding to the experiments made at the Union Workhouse, fully recognize the value of the fluid in destroying offensive smells, but with proper caution add:—"we wish to be understood as pronouncing no opinion respecting the disinfecting powers of the liquor;

* It subsequently oozes out, by the Marquis of Downshire's note, that the fluid is not entitled to the exclusive merit of the cure in the Colonel's cases. The Marquis, writing to him, says—"be so kind as to send some by as early a period as you conveniently can, and write your own directions for its use, and your own *change of diet*, which appears to have so wonderfully brought about the sick under your charge in Dublin and Drogheda."

we have had no opportunity of ascertaining its powers in this respect, the determining of which would require a series of experiments, carefully conducted, and on a large scale."

Should any of our readers wish to learn more respecting the evidence of our Irish brethren about the Ledoyen fluid, they cannot do better than consult the capital article in the last number of our Dublin contemporary, from the pen of the editor. It exposes, with a not-unjust severity, the system of bold and braggart assertion which Colonel Calvert has thought fit to employ, in puffing off the nostrum which he has taken under his immediate protection.

Of the two preparations, which have now been brought before the attention of our readers, we are strongly inclined to believe that Sir W. Burnett's will be found to be most generally and extensively useful. Future experiments, however, can alone decide the point. One thing is certain, viz., that it is much more potently antiseptic than its rival. And this is just what might, *à priori*, be expected; for we know that Chlorine and many of its compounds have a very marked influence on all sorts of decaying matter, as well as upon most, if not all, offensive odours; whereas the action of the Nitrate of Lead appears to be limited to the neutralisation of ammoniacal and hydro-sulphuric gases. Some of our readers may perhaps remember that the late Professor Daniel was led, by the results of some experiments, to suppose that the waters of the estuaries and western sea-coasts of Africa contained a large amount of the last-named gas—generated, it was presumed, by the action of decaying organic matter upon the sulphates contained in the sea-water—and to throw out the hint that the existence of this deleterious gas in the atmosphere might be connected with the production of that pernicious miasm which infests the regions alluded to, and proves so destructive to human life. The accuracy of the above statement has, however, not been confirmed, we believe, by subsequent researches, while the probability of the suggestion is at once set aside by what we know of the development of similar morbific malaria in other parts of the world. No observations, as far as we know, go to prove that there is a fixed connection between the production of any form of fever, and the mere presence of any chemical gas in the atmosphere. The bearing of this remark upon the alleged "disinfecting" properties of the Ledoyen and Burnett fluids, and more especially of the former, will be at once obvious. One remark more, and we have done. Let it never be forgotten that the possession of an efficient antibromic may lead to the very serious evil, in certain circumstances, of getting rid of a temporary nuisance, while the removal of the radical mischief is overlooked or neglected. Dr. Smith and Messrs. Grainger and Toynbee have very properly dwelt, with marked emphasis, upon this point in their report; for, after all, cleanliness and free ventilation are the best sweeteners in the world.

